# Current topic of vascular function in hypertension in 2023–2024

**DOI:** 10.1038/s41440-024-01885-3

**Published:** 2024-09-19

**Authors:** Tatsuya Maruhashi, Yukihito Higashi

**Affiliations:** 1 Department of Regenerative Medicine, Division of Radiation Medical Science, Research Institute for Radiation Biology and Medicine, Hiroshima University, Hiroshima, Japan; 2 Division of Regeneration and Medicine, Medical Center for Translational and Clinical Research, Hiroshima University Hospital, Hiroshima, Japan

**Keywords:** Vascular function, Hypertension, Endothelial function, Arterial stiffness, Brachial-ankle pulse wave velocity, Pulse volume recording

## Abstract

Noninvasive tests of vascular function are useful for assessing the severity of atherosclerosis and risk of cardiovascular events, understanding the pathophysiology of cardiometabolic disorders, and investigating the effects of therapeutic interventions on cardiovascular morbidity and mortality, all of which can provide additional information for the management of patients with cardiovascular risk factors or a history of cardiovascular disease. In 2023–2024, many excellent articles on vascular function were published in Hypertension Research and other major cardiovascular and hypertension journals, and we summarize the emerging evidence on vascular function in this review. We hope that this review will be helpful for the management of patients with cardiovascular risk factors in clinical practice and for future basic and clinical research on vascular function.

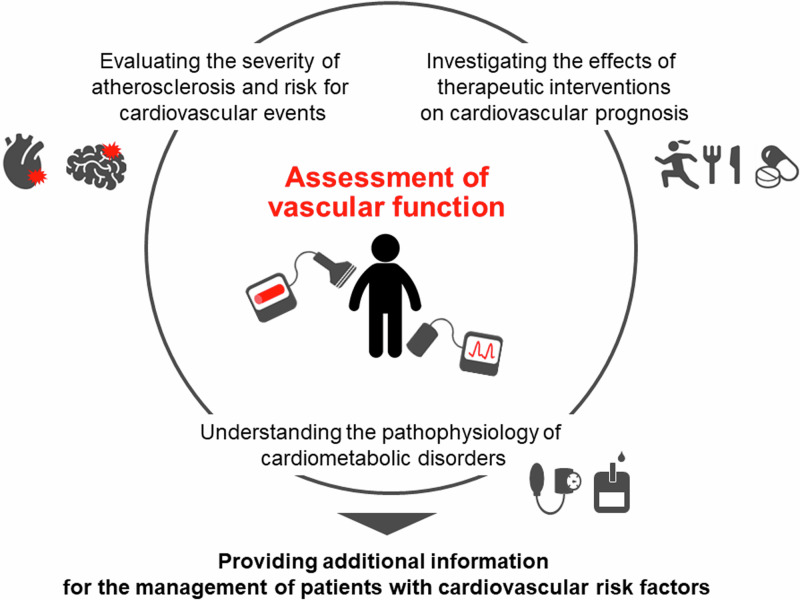

## Introduction

**Fig. 1:**
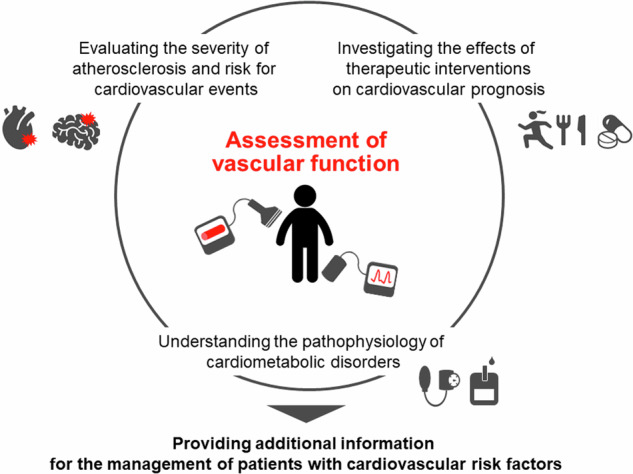
Significance of vascular function tests in clinical practice

Vascular function tests have been used to assess the severity of atherosclerosis and the results of such tests may provide additional information for cardiovascular risk stratification and offer the opportunity for timely intervention to reduce the risk of cardiovascular events in patients with cardiovascular risk factors or a history of cardiovascular events (Fig. 1) (Graphical Abstract) (Table 1) [1, 2]. Assessment of vascular function also provides information for understanding the pathophysiology of cardiometabolic disorders. In addition, vascular function indices may serve as surrogate markers for investigating the effects of therapeutic interventions on cardiovascular morbidity and mortality.

**Table 1:** Advantages and disadvantages of each vascular function test

	Advantage	Disadvantage
Endothelial function		
Flow-mediated vasodilation (FMD)	✓ Repeatable. ✓ A large body of evidence in cardiovascular risk assessment.	✓ Technically challenging. ✓ Significant learning curve for measurements.
Reactive hyperemia peripheral arterial tonometry (RH-PAT)	✓ Easy to use. ✓ Operator-independent. ✓ Standardized measurement method.	✓ Expensive.
Arterial stiffness		
Brachial-ankle pulse wave velocity (baPWV)	✓ Easy to use. ✓ Operator-independent. ✓ Standardized measurement method. ✓ High reproducibility. ✓ A large body of evidence in cardiovascular risk assessment.	✓ Inaccuracy in patients with atrial fibrillation, peripheral arterial disease, and severe aortic valve stenosis.
Cardio-ankle vascular index (CAVI)	✓ Easy to use. ✓ Operator-independent. ✓ Standardized measurement method. ✓ High reproducibility.	✓ Inaccuracy in patients with atrial fibrillation, peripheral arterial disease, and severe aortic valve stenosis.
Stenotic or occlusive lesions		
Pulse volume parameter (upstroke time, percentage mean arterial pressure)	✓ Automatic measurement during ankle-brachial index measurement.	✓ Lack of evidence in cardiovascular risk assessment.

Many excellent articles on vascular function have been published in 2023–2024. This review aims to provide a concise summary of recent literature and new findings regarding vascular function with focus on pathophysiology, cardiovascular risk assessment, and therapeutic intervention. We reviewed the list of articles on vascular function published in Hypertension Research and other major cardiovascular and hypertension journals in 2023–2024, prepared by the editorial office of Hypertension Research. In addition, we conducted our own hand search of articles on vascular function. From these, we selected articles of interest.

## Endothelial function

### Pathophysiology

Cardiovascular risk is strongly influenced by many environmental factors. Among these, the season can have a strong influence on cardiovascular morbidity and mortality. In general, cardiovascular morbidity and mortality are higher in winter than in other seasons. However, it remains unclear whether vascular function is affected by season or outdoor temperature. Maruhashi et al. showed that there was no significant association of flow-mediated vasodilation (FMD) of the brachial artery, an index of endothelial function, or nitroglycerine-induced vasodilation (NID) of the brachial artery, an index of endothelium-independent vasodilation, with season or outdoor temperature, suggesting that it is not necessary to consider season or outdoor temperature when interpreting the results of measurements of FMD and NID when vascular function tests are performed in temperature-controlled room with little fluctuation in the conditions and that vascular function is not associated with seasonal variations in cardiovascular morbidity and mortality [3]. It is possible that the difference in temperature between indoors and outdoors affects vascular function through the autonomic nervous system. However, to our knowledge, there has been no study in which the influence of the difference in temperature between indoors and outdoors on vascular function was investigated. In addition, further studies are needed to determine whether the nonsignificant association of vascular function with season or outdoor temperature can be applied to locations with a markedly different range of outdoor temperatures.

Hyperuricemia may be associated with endothelial dysfunction, and xanthine oxidoreductase (XOR) inhibitors have been shown to improve endothelial function [4]. However, it remains to be determined whether the improvement in endothelial function by XOR inhibitors is due to the reduction of reactive oxygen species (ROS) production or uric acid production. Kurajoh et al. investigated the associations of plasma XOR activity and serum uric acid levels with FMD in 395 subjects without urate-lowering therapy in a cross-sectional study [5]. They showed that plasma XOR activity, but not serum uric acid level, was significantly associated with FMD after adjustment for other confounders and that serum uric acid level was not a mediator of the association of plasma XOR activity with FMD. These findings suggest that XOR contributes to endothelial dysfunction through ROS production, but not uric acid production, and that the improvement in endothelial function by XOR inhibitors may be mainly due to ROS reduction, but not uric acid reduction. The results of this study are inconsistent with the results of previous studies that have shown a significant association between uric acid and endothelial dysfunction in a cross-sectional or prospective study design [6–8]. The exact reasons for this discrepancy are unclear. In the previous studies, XOR activity was not measured. It is possible that the association between uric acid and endothelial dysfunction may become less significant when adjusted for XOR activity. In addition, the median serum uric acid level was 5.4 mg/dL (4.6–6.4 mg/dL) and the prevalence of hyperuricemia (serum uric acid level > 7.0 mg/dL) was 13.4% in this study. Therefore, it may have been difficult to investigate the effect of hyperuricemia on endothelial dysfunction.

Several genetic mutations have been implicated in the pathogenesis of primary aldosteronism. Somatic mutations in KCNJ5 are the most frequently identified genetic mutations in patients with aldosterone-producing adenoma (APA) with incidences of about 40% in Western countries and about 70% in East Asia. KCNJ5 mutation causes excessive aldosterone production by upregulating the expression of CYP11B2, the gene encoding aldosterone synthase, in APA. Kishimoto et al. investigated the associations between *KCNJ5* mutations and changes in vascular function after adrenalectomy in patients with APA [9]. They showed that FMD and NID were significantly impaired in patients with APA compared to those in patients with essential hypertension, regardless of the presence or absence of *KCNJ5* mutation at baseline, and that FMD and NID were significantly improved in APA patients with *KCNJ5* mutation after adrenalectomy, with significant correlations of reductions in plasma aldosterone concentration with improvements in FMD and NID, whereas only NID was improved in APA patients without *KCNJ5* mutation after adrenalectomy. These findings suggest that *KCNJ5* mutation is a predictor of improvement in endothelial function after adrenalectomy in patients with APA. The exact mechanisms underlying the improvement in FMD after adrenalectomy in APA patients with KCNJ5 mutation are unclear. It is generally known that aldosterone levels are higher in APA patients with KCNJ5 mutation than in those without KCNJ5 mutation. Therefore, the large reduction in aldosterone levels with adrenalectomy in APA patients with KCNJ5 mutation may contribute to the improvement in endothelial function after adrenalectomy. However, there was no significant difference in plasma aldosterone concentration before and after adrenalectomy between APA patients with KCNJ5 mutation and those without KCNJ5 mutation. Further studies are needed to investigate the mechanisms between KCNJ5 mutation and endothelial function in patients with APA.

### Risk assessment

Indices of endothelial function may serve as prognostic markers for cardiovascular events. Okawa et al. investigated the association between endothelial function as assessed by the peripheral vascular reactive hyperemia index (RHI) and 5-year cardiovascular events after radiofrequency catheter ablation for atrial fibrillation (AF) [10]. They showed that endothelial dysfunction, defined as an RHI < 2.1 assessed before AF ablation, was significantly associated with cardiovascular events after AF ablation independent of the CHA_2_DS_2_-VASc score. These findings suggest that endothelial function, as assessed by the RHI before AF ablation, can be used as a prognostic marker for cardiovascular events after AF ablation independent of the CHA_2_DS_2_-VASc score in patients with AF. Endothelial dysfunction is the first step in atherosclerosis and is closely associated with the development and progression of atherosclerosis [1]. Therefore, endothelial dysfunction as assessed by RHI may serve as a prognostic marker of cardiovascular events after AF ablation. The association between RHI and AF recurrence was not evaluated in this study. However, Shin et al. showed that baseline endothelial function as assessed by FMD was significantly associated with arrhythmia recurrence after AF ablation [11]. They also showed that FMD was improved in patients with long-term maintenance of sinus rhythm by successful AF ablation. The main mechanism by which endothelial function is improved in patients in sinus rhythm after AF ablation is thought to be that AF-induced irregular turbulent shear stress is restored to regular laminar shear stress, which may improve the expression and activity of endothelial nitric oxide (NO) synthase and consequently NO bioavailability.

Endocan (or endothelial cell-specific molecule-1 [ESM-1]), a soluble dermatan sulfate proteoglycan, is expressed and secreted by the vascular endothelium, particularly during inflammation. Therefore, endocan can be used as a biochemical marker of endothelial function. Behnoush et al. conducted a systematic review and meta-analysis of studies in which serum or plasma endocan levels were investigated in patients with hypertension and healthy controls [12]. They showed that circulating endocan levels were significantly higher in patients with hypertension than in healthy controls, suggesting that endocan can be used as a marker of endothelial function in patients with hypertension. Further studies are needed to determine whether circulating endocan levels correlate with an established marker of endothelial function and whether endocan provides additional information for cardiovascular risk stratification independent of blood pressure in patients with hypertension [13].

### Intervention

Diabetes is associated with endothelial dysfunction, which may lead to the progression of atherosclerosis and the development of cardiovascular events [14]. Glucose-lowering agents are expected to improve endothelial function in patients with diabetes. However, the comparative effects of glucose-lowering agents on endothelial function remain unclear. Kim et al. performed a network meta-analysis to investigate the comparative effects of glucose-lowering agents on endothelial function as assessed by FMD in patients with type 2 diabetes [15]. They showed that sodium-glucose cotransporter-2 (SGLT2) inhibitors, glucagon-like peptide-1 receptor agonists (GLP-1RAs), and thiazolidinediones (TZDs) had significantly more beneficial effects than a placebo on FMD, and that the effects of TZDs on FMD improvement were superior to the effects of glucose-lowering agents including SGLT2 inhibitors and GLP-1RAs. These results support evidence from large clinical trials that SGLT2 inhibitors, GLP-1RAs, and TZDs reduce the risk of cardiovascular events in patients with type 2 diabetes. Insulin resistance is considered to be a major cause of endothelial dysfunction [14]. Although SGLT2 inhibitors and GLP-1RAs are expected to improve insulin resistance through weight reduction, TZDs directly target and specifically treat insulin resistance, which may lead to the superior effect of TZDs on endothelial function compared to SGLT2 and GLP1-RAs.

## Arterial stiffness

### brachial-ankle pulse wave velocity

Recently, brachial-ankle pulse wave velocity (baPWV) has been widely used in clinical practice as a marker of arterial stiffness to assess the severity of atherosclerosis and risk of cardiovascular events. Previously, in the 2018 European Society of Hypertension (ESH) guidelines, only carotid-femoral pulse wave velocity (cfPWV) was indicated as a marker of arterial stiffness for assessment of hypertension-mediated organ damage [16]. However, in the 2023 ESH guidelines for management of arterial hypertension, baPWV was described for the first time as a measure of arterial stiffness along with cfPWV [17].

Although the measurement of baPWV is simple, reproducible, and operator-independent, there are some limitations to its use as a marker of arterial stiffness. The main limitation is that baPWV does not accurately reflect true arterial stiffness in a leg with a low ankle-brachial index (<0.95) because pulse wave velocity may be affected by stenotic or occlusive lesions in lower limb arteries, resulting in lower baPWV values [18]. baPWV may also be affected by body size. Ishida et al. investigated the associations of baPWV with visceral fat area (VFA) as assessed by abdominal computed tomography and several anthropometric indices of obesity, including body mass index, waist circumference, waist-to-height ratio, a body shape index (ABSI), and body roundness index, in 2789 subjects [19]. They showed that VFA and anthropometric indices, except ABSI, were inversely associated with baPWV. These results suggest that baPWV improves with increasing body weight. Although the precise mechanisms underlying the negative association between BMI and baPWV are unclear, one possible explanation is that larger arterial diameter in obese subjects than in lean subjects contributes to the negative association between BMI and baPWV. Since PWV decreases as vessel diameter increases, larger arterial diameter may be associated with lower PWV in obese subjects, which can partially explain the negative association between BMI and baPWV. However, it remains to be elucidated whether a lower value of baPWV in obese subjects truly reflects the protective effect of obesity on arterial stiffness (obesity paradox) and whether general cutoff values of baPWV for cardiovascular risk assessment (<1400 cm/s for low risk, 1400–1800 cm/s for intermediate risk, and >1800 cm/s for high risk) can be applied to obese subjects [2].

baPWV may also be affected by body position during measurement. Alhalimi et al. showed that baPWV gradually increased with increasing upper body angle during measurement and that the increase in baPWV became statistically significant when the body position was tilted upward by 10° or more [20]. In contrast, knee flexion decreased baPWV at any upper body angle. These results indicate the importance of measuring baPWV in the correct supine position for accurate assessment of arterial stiffness.

### Pathophysiology

Increased arterial stiffness may exert deleterious effects on the heart by increasing the cardiac afterload and decreasing coronary blood flow. Yatsu et al. investigated overnight changes in arterial stiffness as assessed by the cardio-ankle vascular index (CAVI) and the association between overnight increase in CAVI and sleep-disordered breathing in 60 patients hospitalized for acute heart failure (HF) [21]. They showed that CAVI significantly increased overnight and that moderate-to-severe sleep-disordered breathing and obstructive respiratory events were significantly associated with an overnight increase in CAVI, suggesting that obstructive sleep apnea, rather than central sleep apnea, is associated with an overnight increase in CAVI. These findings suggest that sleep-disordered breathing may adversely affect patients with acute HF through an overnight increase in arterial stiffness. During the acute phase of HF, the fluid shift may predispose to obstructive sleep apnea, which in turn may lead to an overnight increase in CAVI, i.e., increased aortic stiffness. The increased aortic stiffness increases left ventricular afterload, thereby forming a vicious cycle between obstructive sleep apnea and acute HF.

The inter-arm systolic blood pressure difference (IAD) has been shown to be a significant predictor of cardiovascular events [22]. Shiina et al. investigated the association between obstructive sleep apnea (OSA) and IAD in 2643 consecutive subjects who underwent polysomnography. They showed that OSA was independently associated with an IAD ≥ 10 mmHg, suggesting that OSA contributes to structural changes in the thoracic aorta through negative intrathoracic pressure [23]. The presumed mechanism of the association between IAD and OSA is that the negative intrathoracic pressure generated in patients with OSA is associated with aortic remodeling, aortic dissection, aortic aneurysm, and increased thoracic aorta diameter, resulting in IAD [23].

Tomiyama et al. investigated the association between lifelong status of each cardiovascular risk factor and the rate of progression of arterial stiffness as assessed by baPWV in 3763 untreated subjects over a 16-year period [24]. The authors showed that smoking, heavy alcohol intake, diabetes, hypertension, hypertriglyceridemia, and hyperuricemia were independently associated with rapid progression of baPWV, whereas body mass index and low-density lipoprotein cholesterol were not associated with the progression of baPWV.

Ishida et al. investigated the associations of baPWV and blood pressure status with cerebral small vessel disease (CSVD), including white matter hyperintensities, cerebral microbleeds, silent lacunar infarcts, and enlarged perivascular spaces, in a cross-sectional study of 1894 stroke-free participants who underwent brain magnetic resonance imaging and baPWV measurements at a health checkup [25]. The authors showed that a high baPWV (≥14.63 m/s) was significantly associated with a higher risk of CSVD independent of blood pressure status and that the association between baPWV and CSVD was stronger in participants younger than 60 years than in participants ≥60 years. These results suggest that higher arterial stiffness may be a more important contributor to CSVD than blood pressure status in stroke-free individuals, particularly in younger individuals. The associations between baPWV and each subtype of CSVD were not evaluated in this study.

### Risk assessment

Zhen et al. investigated whether arterial stiffness as assessed by baPWV was independently associated with new onset of HF in 40,064 participants without a history of HF or AF, with a mean follow-up period of 5.53 years [26]. The authors showed that participants with borderline baPWV (1400≤baPWV<1800 cm/s) and participants with elevated baPWV (≥1800 cm/s) had a significantly higher risk of incident HF than did participants with normal baPWV (<1400 cm/s), suggesting that arterial stiffness contributes to the development of HF and that baPWV can be used as a prognostic marker for HF.

For cardiovascular health (CVH), the American Heart Association proposed the use of a composite of ideal CVH markers for the prevention of cardiovascular disease, called Life’s Essential 8 (LE8), which includes eight components of CVH: healthy diet, participation in physical activity, avoidance of nicotine, healthy sleep, healthy weight, and healthy levels of blood lipids, blood glucose, and blood pressure [27]. Wu et al. investigated whether the CVH score calculated from the LE8 metrics predicts baPWV progression and stroke risk in 68,885 subjects [28]. The authors showed that a better CVH score was associated with lower baPWV at baseline, slower progression of baPWV, and lower risk of stroke events during a median follow-up period of 5.65 years and that 9.07% of the total association between CVH score and incident stroke was mediated by baPWV. These results suggest that the CVH score, as assessed by EL8 metrics, may serve as a prognostic marker for baPWV progression and incident stroke and that adherence to a healthy lifestyle and maintaining metabolic parameters within normal ranges may prevent arterial stiffening and stroke events.

## Other parameters

High-quality pulse volume waveforms of the brachia and ankles can be obtained automatically in a short time during ankle-brachial index (ABI) measurement using a volume-plethysmographic apparatus (Form PWV/ABI, Omron Health Care Co., Kyoto, Japan). The device automatically calculates pulse volume recording (PVR) parameters, including upstroke time (UT), transit time from the nadir to the peak of the pulse wave, and percentage mean arterial pressure (%MAP), the percentage of enclosed pulse volume area relative to the rectangle with the pulse’s base and amplitude, of each limb and displays these parameters immediately after ABI measurements, leading to the clinical use of UT and %MAP in combination with ABI [29]. Pulse waveforms in the limbs were recorded and stored for 10 s. The upstroke time and %MAP were measured for each pulse waveform, and the means of the upstroke times and %MAPs obtained in the 10-s recording were automatically calculated. Pulse volume waveforms from limbs with stenotic or occlusive arterial lesions are characterized by an absence of a dicrotic notch, a prolonged systolic downslope, a round systolic peak, and a flattened wave, resulting in prolonged upstroke time and increased %MAP [29]. Recent clinical studies have shown that both UT and %MAP at the ankle may serve as cardiovascular risk markers [30, 31]. However, there is little information on whether UT or %MAP is more useful for cardiovascular risk assessment in individuals with a normal ABI. Maruhashi et al. investigated the optimal cutoff values of UT and %MAP for identifying patients with clinical CAD and directly compared the diagnostic accuracy of UT and that of %MAP for clinical CAD in individuals with a normal ABI [32]. They showed that the optimal cutoff values of UT and %MAP for diagnosing clinical CAD obtained from receiver operating characteristic (ROC) curve analyses were 148 ms and 40.4%, respectively, which were much lower than the recommended cutoff values of 180 ms for UT and 45.0% for %MAP for screening lower extremity arterial disease, and that the area under the curve value of UT was significantly higher than that of %MAP. In addition, the addition of UT, but not %MAP, to the traditional risk factors improved the diagnostic accuracy for clinical CAD. These findings suggest that UT is more useful than %MAP for identifying patients with clinical CAD in individuals with a normal ABI. More attention should be paid to PVR parameters, especially UT at the ankle, to identify patients with clinical CAD, which may reduce the risk of missing patients with a high cardiovascular risk in individuals with a normal ABI.

## Conclusions

In this article, we reviewed the recent literature and summarized new findings on vascular function. Vascular function tests are valuable for obtaining a better understanding of the pathophysiology of cardiometabolic diseases, for more accurate cardiovascular risk assessment, and for evaluating the effects of therapeutic interventions on atherosclerosis. Further accumulation of data on vascular function will allow us to draw more specific conclusions regarding the role of vascular function in cardiovascular disease.

## Future perspectives

Patients at a high risk for cardiovascular events based on the status of traditional cardiovascular risk factors and the results of vascular function tests are intensively managed using blood pressure, lipid parameters, and blood glucose levels as indicators. However, there are no data showing that therapeutic interventions based on the results of vascular function tests have improved cardiovascular prognosis. Further studies are needed to determine whether therapeutic interventions using vascular function as a therapeutic index improve cardiovascular prognosis compared with interventions using traditional cardiovascular risk factors such as blood pressure, lipid parameters, and blood glucose as therapeutic indices.
